# Ring 1 and YY1 Binding Protein is Expressed in Murine Spermatocytes but Dispensable for Spermatogenesis

**DOI:** 10.3390/genes11010084

**Published:** 2020-01-11

**Authors:** Zhen He, Rong-Ge Yan, Xiao-Na Zhang, Qi-En Yang

**Affiliations:** 1 Key Laboratory of Adaptation and Evolution of Plateau Biota, Northwest Institute of Plateau Biology, Chinese Academy of Sciences, Xining 810001, China; 2 University of Chinese Academy of Sciences, Beijing 100049, China; 3 Qinghai Key Laboratory of Animal Ecological Genomics, Northwest Institute of Plateau Biology, Chinese Academy of Sciences, Xining 810001, China

**Keywords:** Rybp, spermatogenesis, meiosis, germ cells, polycomb group 1

## Abstract

Spermatogenesis is a complex cellular-differentiation process that relies on the precise regulation of gene expression in spermatogonia, meiotic, and postmeiotic germ cells. The Ring 1 and YY1 binding protein (Rybp) is a member of the mammalian polycomb-group (PcG) protein family that plays multifunctional roles in development. Previous findings indicate that Rybp may function as an important regulator of meiosis. However, its expression in the testes and function in spermatogenesis have not been examined. In this study, we investigated Rybp expression in postnatal mouse testes using qRT-PCR and immunohistochemistry. We also examined the function of Rybp in spermatogenesis by using a conditional-knockout approach. Results showed that the relative expression of *Rybp* mRNA was significantly upregulated in the testes of postnatal day (PD) 6 mice. Immunofluorescent staining revealed that Rybp was enriched in the spermatocytes. Surprisingly, a conditional deletion of *Rybp* in fetal germ cells did not affect the fertility or normal development of spermatogenic cells. Further analysis revealed that *Rybp* deletion resulted in a decreased expression of meiosis-related genes, but that meiosis progression was normal. Together, these findings suggest that Rybp expression was enriched in spermatocytes, but that it was not required for spermatogenesis.

## 1. Introduction

Spermatogenesis is a complex differentiation process that includes three different cellular events—spermatogonial mitosis, spermatocyte meiosis, and spermiogenesis [1]. Meiosis is a germ-cell-specific event that is essential for generating new allelic combinations through recombination [2]. Programs regulating gene expression in germ cells must be tightly controlled to ensure the initiation, progression, and completion of meiosis [3]. Currently, the molecular basis of meiosis initiation and progression is poorly understood in mammals.

Polycomb-group (PcG) proteins play important roles in establishing and maintaining gene-expression patterns during cell differentiation and proliferation [4]. PcG proteins form two polycomb repressive complexes, PRC1 and PRC2 [5]. PRC1 includes a chromobox (Cbx2, Cbx4, Cbx6, Cbx7, or Cbx8), polyhomeotic-like protein (PHC1, PHC2, or PHC3), very interesting new gene 1A/B (Ring1A/B), polycomb group RING finger proteins (PCGF1, PCGF2, PCGF3, PCGF4, PCGF5, or PCGF6), and YY1-associated factor 2 (YAF2) [6,7]. PRC1 possesses ubiquitin E3 ligase activity that targets H2AK119. This modification usually induces chromatin compaction and inhibits transcriptional elongation. Recent studies showed that it can also activate gene expression [8]. Accumulated evidence has revealed indispensable roles for PRC1 in spermatogenesis. Sex comb on midleglike 2 (SCML2), a germline-specific subunit of PRC1, acts in concert with histone H2A ubiquitination to regulate the postmeiotic development of male germ cells. Deletion of *Scml2* results in the accumulation of ubiquitinated H2AK119 and the dysregulation of postmeiotic genes [9]. RING finger protein 2 (RNF2, also known as RING1B) is required for meiosis, and *Rnf2*-deficient germ cells arrest at the midpachytene stage [10].

Other components of PRC1 also play different roles in meiosis. MYC family proteins (MAX) are newly recognized regulators of meiosis initiation. A loss of *Max* in embryonic stem (ES) cells activates germ-cell-related genes and leads to cytological changes resembling the leptotene and zygotene stages of meiosis [11]. Polycomb group RING finger 6 (PCGF6) is predominantly expressed in spermatocytes and spermatids. It interacts with heat shock-related 70-kDa protein 2 (HSPA2), which is an essential factor in male meiosis [12]. Chromobox homolog protein 2 (Cbx2) plays a critical role in germ-cell viability, meiosis initiation, and homologous chromosome synapsis in the mammalian germline [13]. Functional roles of other PRC1 members in spermatogenesis remain unexplored.

Ring 1 and YY1 binding protein (Rybp) is a noncanonical PRC1 component that serves multifunctional roles in development [6]. Loss of Rybp causes marked forebrain overgrowth, a disruption of neural-tube closure, retinal coloboma, malformed lenses, and a failure to form contractile cardiomyocytes [14,15,16,17]. Interestingly, several lines of evidence indicated that Rybp has a potentially crucial role in germ-cell development and meiosis. Rybp can efficiently repress endogenous retroviruses and germline-specific genes [18]. *Dazl*, *Rhox6*, *Ddx4*, *Tex11*, and *Mov10l1* are genes that regulate germ-cell differentiation and meiosis, and they are significantly upregulated in *Rybp*-mutant ES cells [18]. These data strongly suggest that Rybp may function as an important transcriptional regulator in meiosis. Recently, Bajusz et al. proposed that a Rybp-dependent transcriptional program is important for germ-cell differentiation [19]. However, the expression and function of Rybp in spermatogenesis have not been explored. 

In this study, we investigated the dynamics of Rybp expression in murine testes using qRT-PCR and immunohistochemistry. We also conditionally deleted *Rybp* in germ cells using Cre–Loxp methodology in order to examine the function of Rybp in meiosis. Our results showed that, although Rybp was expressed in murine spermatocytes and the expression of several meiosis-related genes was significantly reduced in *Ddx4-Cre^+^*;*Rybp^flox/flox^* (designated hereafter as *Rybp*-cKO) mice, Rybp was not required for spermatogenesis. 

## 2. Materials and Methods

### 2.1. Generation of Germ-Cell-Specific Rybp-Knockout Mice

All animal studies were performed in accordance with guidelines from the Institutional Animal Care and Use of Laboratory Animals, and were approved by the Animal Welfare and Ethic Committee at the Northwest Institute, Chinese Academy of Sciences (approval code: hwipb012). *Rybp^flox/flox^* mice (T00008, B6; 129-*Rybp^tm1Nju^*) were generated by the Nanjing Biomedical Research Institute of Nanjing University, and *Ddx4-Cre* mice were obtained from the Jackson Laboratory (018980, B6; FVB-Tg (*Ddx4-Cre*) 1Dcas/Knwj). *Rybp^flox/flox^* females were mated with *Ddx4-Cre^+^* males to generate *Ddx4-Cre^+^*;*Rybp^flox/+^* males and *Rybp^flox/+^* females. *Ddx4-Cre^+^*;*Rybp^flox/+^* males were then mated with *Rybp^flox/flox^* or *Rybp^flox/+^* females to obtain *Ddx4-Cre^+^*;*Rybp^flox/flox^* (*Rybp*-cKO) males and *Ddx4-Cre^+^*;*Rybp^flox/+^* males (controls). The tip of each mouse tail was used for genotyping. Primers used to detect the *Rybp*-flox allele are listed in Table S1—the intact allele was 318 base pairs (bp) and *Rybp*-flox allele was 421 bp. The primers used to detect *Ddx4-Cre* are listed in Table S1—the *Ddx4-cre* allele was 240 bp and the wild type (WT) allele was 324 bp.

### 2.2. Quantitative RT-PCR

RNA isolation and quantitative RT-PCR were performed as previously described [20]. RNA samples were isolated using a Trizol reagent (Invitrogen, Carlsbad, CA, USA). RNA concentration and purity were quantified using a Nanodrop 2000c Spectrophotometer (Thermo, Waltham, MA, USA), and RNA was reverse transcribed using a High-Capacity cDNA Reverse Transcription kit (Applied Biosystems, Foster, CA, USA). An SYBR Green Detection System was used in combination with primer pairs (10 μM; Table S2). A ViiA7 Real-Time PCR System (Applied Biosystems, Foster, CA, USA) was used to quantify the relative abundance of specific transcripts. The optimized parameters for thermal cycles were as follows: activation at 95 °C for 2 min, followed by 40 cycles consisting of 95 °C for 20 s and 60 °C for 30 s. The temperature was then gradually increased (0.5 °C/s) to 95 °C to generate the melting curve. In this experiment, GAPDH was used as the internal parameter for quantitative results. The experiment was repeated 3 times for each sample with 3 biological duplicates for each gene; mRNA expression levels were calculated using the 2^−ΔΔct^ method.

### 2.3. Histological Analysis

Histological analysis of testicular sections was performed as previously described [21]. Briefly, mouse testes were fixed in Bouin’s solution for 8 h. After dehydration, tissue samples were embedded in paraffin (Leica, Mannheim, Germany). Paraffin-embedded tissue was then cut into 4 μm slices by a microtome (Leica RM2235, Mannheim, Germany). Sections were deparaffinized, rehydrated, and stained with hematoxylin and eosin (H&E). Images were examined using a microscope (Nikon ECLIPSE E200, Tokyo, Japan) and captured by Charge Coupled Device (CCD) (MshOt MS60, Guangzhou, China).

### 2.4. Immunohistochemical Staining

Testes were fixed in 4% paraformaldehyde (PFA). After dehydration, tissue samples were embedded in paraffin. Paraffin-embedded tissue was cut into 4 µm slices by a microtome. After deparaffinization and rehydration, sections were boiled in 10 mM sodium citrate (pH 6.0) for 20 min and washed in 0.01 M phosphate-buffered saline (PBS) for 5 min. This was repeated 3 times at room temperature (RT). Endogenous peroxidase activity was blocked by 3% H_2_O_2_ for 10 min at RT. Sections were sequentially washed 3 times and incubated with 10% normal goat serum for 1 h at RT. Primary antibodies (Table S3) were diluted in an antibody dilution buffer and incubated overnight at 4 °C. Sections were then washed in PBS and incubated with secondary antibodies for 1 h at RT. After being washed 3 times in PBS for 10 min each time, sections were visualized using 3,3-diaminobenzidine (DAB, ZSGB-BIO, Beijing, China) and counterstained with Ehrlich’s hematoxylin. For immunofluorescent staining, sections were incubated with 10% normal donkey serum for 1 h at RT. After incubation with primary antibodies, sections were washed and incubated with secondary antibodies (Table S3) for 2 h at RT. After being washed 3 times in PBS for 10 min each time, sections were stained with Hoechst33342 (H33342) (Sigma, St. Louis, MO, USA) for 1 min and mounted in 50% glycerol before being examined under a microscope (Leica, Mannheim, Germany). 

### 2.5. Fertility Test

The fertility of males with genotypes *Ddx4-Cre^+^*; *Rybp^flox/flox^* (*Rybp*-cKO) and *Ddx4-cre^+^*; *Rybp^flox/+^* (controls) was assessed by mating with 4 adult *Rybp^flox/+^* females. This began at 35 days. They mated until the male mice were 3 months old in all 3 groups. Males were then sacrificed, and their testes and body weight were measured. Cauda epididymides were placed in 1 mL human tubal fluid (HTF) (Merck Millipore, MA, USA), cut into pieces, then put on ice for 10 min to fully release the sperm. Sperm was then counted by a computer-assisted sperm-analysis system (Ningbo Shengheng Optics and Electronics Co., Ltd., Ningbo, China).

### 2.6. Chromosome Spreads of Mouse Spermatocytes and Staining

Chromosome spreads of spermatocytes from 21 day old *Rybp*-cKO and control mice were performed as previously described [22]. Briefly, seminiferous tubules were transferred to a hypotonic buffer (30 mM Tris-HCl at pH 8.2, 17 mM sodium citrate, 5 mM ethylenediaminetetraacetic acid, 50 mM sucrose, 5 mM dithiothreitol, and 0.5 mM phenylmethylsulfonyl fluoride) for 30 min at RT. Sucrose (100 mM) was dropped onto a clean slide. Seminiferous tubules were mixed with the sucrose drop on the clean slide and then disrupted to obtain cell suspension, which was then spread over the slide immersed in 1% PFA. Adhesive slides with the cell suspension were put into a chamber with hot water (90–100 °C at normal atmospheric pressure) overnight at RT. Slides were washed in Antibody Dilution Buffer (ADB) (0.1% cold fish skin gelatin, 0.5% Tritonx-100, and 1% bovine serum albumin (BSA) in PBS) for 1 h the following day. A combination of primary antibodies (Table S3) that had been diluted in ADB and incubated overnight at 37 °C in a wet chamber was added. After being washed with ADB for 30 min, we washed the slides with a new ADB for 90 min and incubated the slides with secondary antibodies (Table S3) overnight at 37 °C. We then washed the slides in ADB for 30 min and PBS for 1 h. Slides were exposed to H33342 for 1 min and mounted in 50% glycerol before being examined under a microscope (Leica, Mannheim, Germany). 

### 2.7. Statistical Analysis

All quantitative data are presented as mean ± Standard Error of Mean (SEM) for at least 3 biological replicates. Differences between means were examined using the general linear-model one-way ANOVA or *t*-test function of GraphPad Prism 5 (La Jolla, CA, USA). Differences between means were considered significant when *p* < 0.05.

## 3. Results

### 3.1. Relative mRNA Expression and Protein Localization of Rybp in Postnatal Mouse Testes

**Figure 1:**
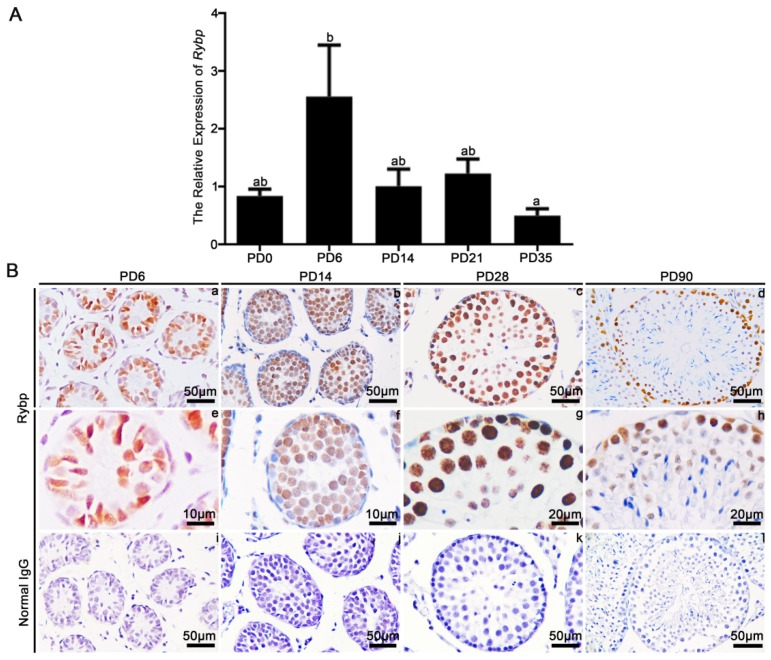
Relative mRNA expression and protein localization of Ring 1 and YY1 binding protein (Rybp) in murine testes. (**A**) Quantification of *Rybp* mRNA expression in murine testes at different stages of development. Data were analyzed using mean ± SEM for three mice per stage. Values bearing different superscripts significantly differed with *p* < 0.05. (**B**) Immunohistochemical staining of Rybp in murine testes at different stages of development. Scale bar: a–d and i–l, 50 µm; e and f, 10 µm; g and h, 20 µm.

First, we examined the expression of *Rybp* mRNA in the testes of mice at postnatal day (PD) 0, 6, 14, 21, and 35 using qRT-PCR. Results showed that the expression of the *Rybp* transcript was significantly upregulated in the testes of mice at PD6 (*p* < 0.05; Figure 1A). We then examined the expression and cellular localization of Rybp in testes using immunohistochemistry (Figure 1B). Results showed that Rybp was localized to Sertoli cells and spermatogonia at PD6, and was present in spermatocytes at PD14. The staining signal was also seen in spermatids at PD28 and PD90. In adult testes, the Rybp signal was distributed between spermatogonia, spermatocytes, and Sertoli cells (Figure 1B). The negative control with normal IgG did not show an immunoreactive signal (Figure 1B). 

**Figure 2:**
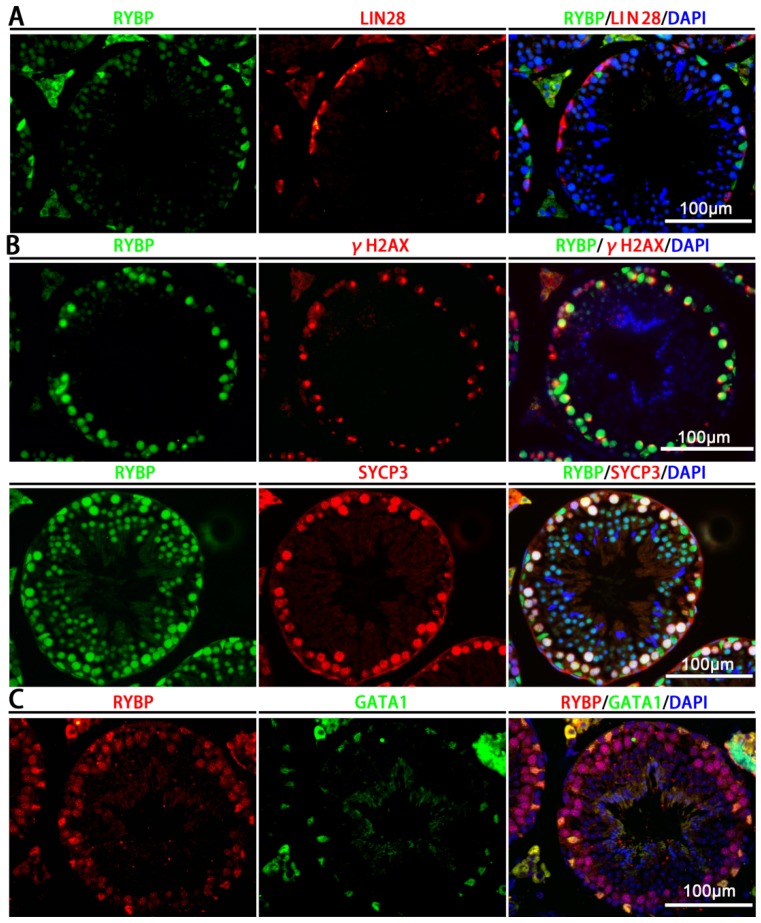
Rybp expressed in spermatocytes and Sertoli cells. (**A**) Coimmunofluorescent staining for Rybp and LIN28A in cross-sections of testes from adult mice. Scale bar, 100 µm. (**B**) Immunofluorescent staining for Rybp, γH2AX, and synaptonemal complex protein 3 (SYCP3) in cross-sections of testes from adult mice. Scale bar, 100 µm. (**C**) Immunofluorescent staining for Rybp and GATA1 (Sertoli cell marker) in cross-sections of testes from adult mice. Scale bar, 100 µm.

To confirm the findings of the immunohistochemical staining, we costained Rybp with undifferentiated spermatogonial marker LIN28A [23], meiotic germ-cell markers γH2AX and synaptonemal complex protein 3 (SYCP3) [24], and Sertoli cell marker GATA1 [25]. Results showed that Rybp did not colocalize with LIN28A in undifferentiated spermatogonia (Figure 2A). A strong immunostaining signal for Rybp was seen in γH2AX- and SYCP3-positive germ cells (Figure 2B). GATA1^+^ Sertoli cells also stained positive for Rybp (Figure 2C). Together, these data suggest that Rybp was expressed in both Sertoli cells and spermatocytes. 

### 3.2. Phenotypic Analyses of Germ-Cell-Specific Rybp-Knockout Mice

**Figure 3:**
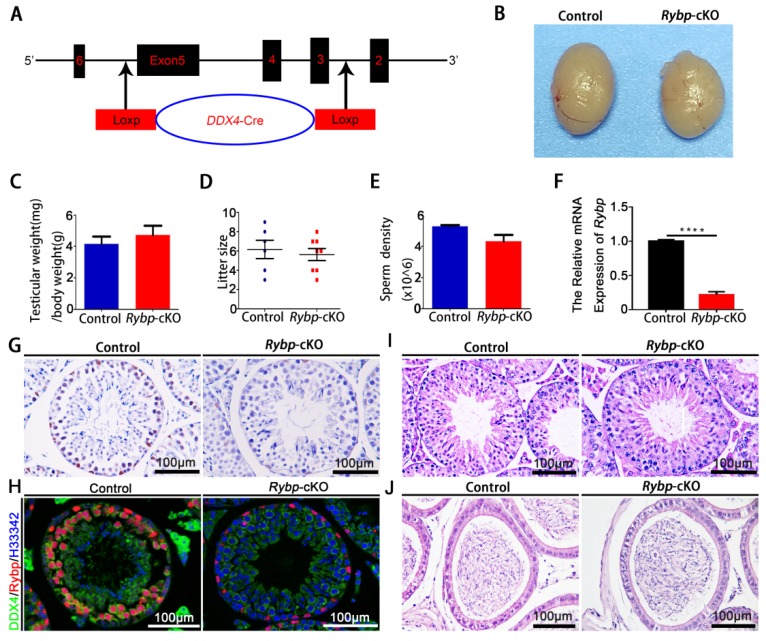
Germ-cell-specific *Rybp*-knockout mice were fertile. (**A**) Schematic diagram of *Ddx4-Cre^+^*;*Rybp^flox/flox^* (*Rybp*-cKO) mice generated using Cre–Loxp technology with exons 2–6. (**B**) Representative images of testes from three-month-old male control and *Rybp*-cKO mice. (**C**) Ratios of testes to body weight of three-month-old male control and *Rybp*-cKO mice. (**D**) Comparisons of litter size from three-month-old male control and *Rybp*-cKO mice. (**E**) Comparisons of sperm density from male control and *Rybp*-cKO mice. (**F**) Quantification of *Rybp* mRNA expression in testes from three-month-old male control and *Rybp*-cKO mice. Data were analyzed using mean ± SEM for three mice per genotype. **** indicates a significant difference of *p* < 0.0001. (**G**) Immunohistochemical staining for Rybp in cross-sections of testes from three-month-old male control and *Rybp*-cKO mice. Scale bar, 100 μm. (**H**) Coimmunofluorescent staining for Rybp and DEAD-box helicase 4 (DDX4) (a germ-cell marker) in cross-sections of testes from three-month-old male control and *Rybp*-cKO mice. Scale bar, 100 μm. (**I**) Representative images of hematoxylin and eosin (H&E)-stained testes from three-month-old male control and *Rybp*-cKO mice. Scale bar, 100 μm. (**J**) Representative images of H&E-stained cauda epididymides from three-month-old male control and *Rybp*-cKO mice. Scale bar, 100 μm. Data were analyzed using mean ± SEM for three mice per genotype.

To explore whether Rybp plays a functional role in germ-cell differentiation and meiosis, we conditionally deleted *Rybp* in fetal gonocytes on embryonic day (E) 14.5 by crossing *Ddx4-Cre* transgenic mice and *Rybp^flox/flox^* mice. Surprisingly, we found that the testes/body-weight ratio of three-month-old *Ddx4-Cre^+^*;*Rybp^flox/flox^* (*Rybp*-cKO) mice did not differ from age-matched littermate controls (*Ddx4-Cre^+^*;*Rybp^flox/+^*) (Figure 3B,C). Fertility testing revealed that control and *Rybp*-cKO mice sired a similar number of litters after mating with wildtype females for three months (Figure 3D). As expected, sperm density in control and *Rybp*-cKO mice was not different (Figure 3E). Further analysis confirmed that Rybp was completely eliminated from the germ cells of *Rybp*-cKO mice (Figure 3F–H), although histological analysis of seminiferous tubules and cauda epididymides revealed normal spermatogenesis (Figure 3I,J). These findings support the conclusion that Rybp is not required for normal fertility and spermatogenesis in mice. 

### 3.3. Analysis of Meiosis Progression and Meiosis-Related Gene Expression in Germ-Cell-Specific Rybp-Knockout Testes

**Figure 4:**
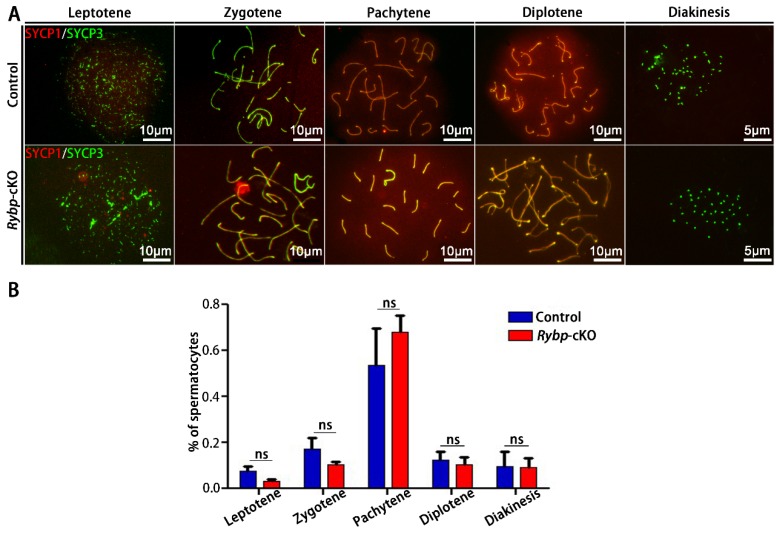
Germ-cell-specific deletion of *Rybp* did not affect meiosis. (**A**) Coimmunostaining of SYCP3 and SYCP1 in spermatocytes from male postnatal day 21 (PD21) control and *Rybp*-cKO mice. (**B**) Composition of the spermatocyte population in control and *Rybp*-cKO spermatocytes. Data were analyzed using mean ± SEM for three mice per genotype. ns: indicates no difference.

Given that the target genes of Rybp in ES cells predominantly regulate the M phase of meiosis [6], we examined meiosis progression and the expression of several previously identified Rybp target genes in control and *Rybp*-cKO mice. The meiotic chromosome spreading and immunostaining of synaptonemal complex proteins SYCP3 (axial/lateral element) and SYCP1 (central element) were performed to examine the progression of meiosis [26]. Results showed that spermatocytes in control and *Rybp*-cKO mice had no apparent synaptic defects at different stages of prophase I (Figure 4A). The percentage of germ cells in leptene, zygotene, pachytene, diplotene, and diakinesis was comparable between control and *Rybp*-cKO testes (Figure 4B).

**Figure 5:**
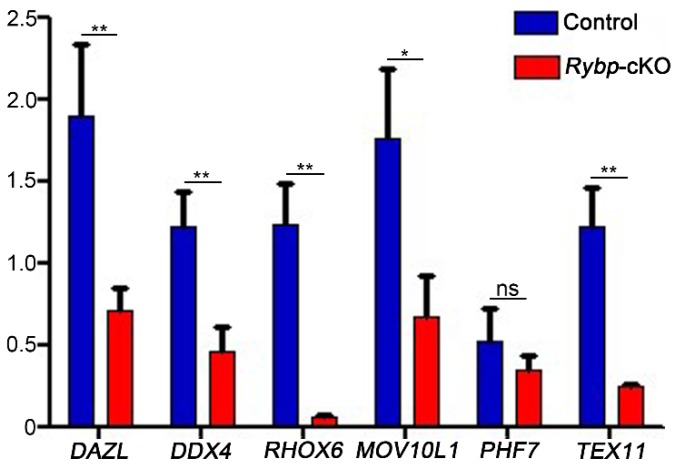
Expression of meiosis-related genes decreased in germ-cell-specific *Rybp*-knockout mice. Relative mRNA expression of meiosis-related genes in male control and *Rybp*-cKO mice. Data were analyzed using mean ± SEM for three mice per group. * indicates significant difference of *p* < 0.05. ** indicates extremely significant difference of *p* < 0.01. ns indicates no difference.

Interestingly, the relative abundance of transcripts known to be important in meiosis [18] was changed by a loss of Rybp function in germ cells. *Dazl*, *Rhox6*, *Ddx4*, *Tex11,* and *Mov10l1* were downregulated by 61.21%, 62.29%, 95.94%, 61.36%, and 80.32%, respectively, in *Rybp*-cKO testes compared to control testes (Figure 5). The relative expression of *Pfh7* did not differ between control and *Rybp*-cKO mice (Figure 5). Together, these findings suggest that *Rybp* deletion in germ cells altered the expression of several meiosis-related genes, but did not impact meiosis progression. 

## 4. Discussion

Rybp is a component of PRC1 that has indispensable roles in the development of several cell lineages. Previous findings indicated that it may have an important role in germ-cell development and meiosis. In this study, we found that Rybp was expressed in spermatocytes and Sertoli cells in mice. To analyze its functional role in spermatogenesis, we conditionally deleted *Rybp* in germ cells. Surprisingly, we found that, although the deletion of *Rybp* in fetal germ cells altered the expression of several meiosis-related genes, loss of Rybp function did not influence normal spermatogenesis. 

An interesting finding of the present study was that the deletion of *Rybp* in germ cells did not impact normal spermatogenesis. Cre activity in *Ddx4-Cre* transgenic mice was detected in germ cells as early as E14.5 [27], and we confirmed that Rybp expression was efficiently removed from all germ cells in *Rybp*-cKO males. Therefore, we concluded that Rybp was not required for spermatogonial differentiation and meiosis. However, the function of Rybp in germ-cell specification and early germ-cell development remains undetermined. Germ-cell specification occurs around E6.25 in mice, and primordial germ-cell migration to the genital ridges occurs by E10.5. During this period of development, germ cells undergo extensive epigenetic reprogramming [28]. From E12.5 to E14.5, pluripotent genes are downregulated, and germ cells differentiate into gonocytes [29]. Given Rybp is a potent regulator of early-lineage commitment [17], we speculate that Rybp may have a functional role in lineage specification to regulate the fate of primordial germ cells. 

In addition to its role in the repression of meiosis-specific gene expression, Rybp is a component of homologous recombination repair machinery, which is important for mitosis and meiosis [30]. Therefore, it was surprising to find that *Rybp* deletion did not affect meiosis progression. YY1-associated factor 2 (YAF2) can rescue the phenotype caused by a *Rybp* mutation [31]; thus, it is likely that YAF2 compensates for the loss of Rybp in spermatocytes. One key function of Rybp that is independent of YAF2 is the regulation of H2AK119ub1 and PRC1 [32]. We concluded from the present study that Rybp-dependent H2AK119ub1 regulation is not required for spermatogenesis, but that Rybp/YAF2-depedent PRC1 activity may still be important for meiosis and other crucial events during germ-cell development. 

Although meiosis was not disrupted by *Rybp* deletion in fetal germ cells, the expression of several meiosis-related genes was downregulated. We examined Rybp-regulated genes in ES cells [18] and found that the relative abundance of all five transcripts (except *Pfh7*) was decreased. Deleted in azoospermia-like (Dazl) and DEAD-box helicase 4 (Ddx4) are RNA-binding proteins that play essential roles in primordial germ-cell migration, spermatogonial differentiation, and meiosis [33,34]. In ES cells, Rybp represses the expression of these germ-cell-specific transcripts by regulating DNA methylation [18]. In spermatocytes, it appears that Rybp stimulates the expression of these genes. A two-hybrid screen identified that Rybp interacts with transcription factors E2F2 and E2F3 to activate gene expression [35], and a similar mechanism likely exists in spermatocytes. However, this regulatory machinery is not required for meiosis progression and the development of advanced germ cells. 

## 5. Conclusions

We showed that Rybp is expressed in spermatocytes and Sertoli cells in murine testes. Although previous findings suggest an important role for Rybp in germ-cell differentiation and meiosis, conditional deletion of *Rybp* in fetal germ cells caused decreased expression of germ-cell-specific genes, but did not affect fertility. Further analysis showed that the development of spermatogenic cells and meiosis were normal in *Rybp* germ-cell-specific conditional-knockout mice.

## Data Availability

The authors declare that the data supporting the findings of this study are available within the paper and its supplementary materials, or are available from the corresponding author upon reasonable request.

## Supplementary Materials

The following are available online at https://www.mdpi.com/2073-4425/11/1/84/s1. Table S1: PCR-related primer list, Table S2: Real-time PCR-related primer list, Table S3: Antibody Information in this article.

## Abbreviations

Rybp	Ring 1 and YY1 binding protein
PcG	Polycomb group
PRC1	Polycomb repressive complex 1
PRC2	Polycomb repressive complex 2
qRT-PCR	Quantitative real-time PCR
H&E	Hematoxylin and eosin
PBS	Phosphate-buffered saline
DAB	3,3-diaminobenzidine
RT	Room temperature
H33342	Hoechst33342
PFA	Paraformaldehyde
BSA	Bovine serum albumin
bp	Base pair(s)
PD	Postnatal day
