# Supplementary material for: Ring 1 and YY1 Binding Protein is Expressed in Murine Spermatocytes but Dispensable for Spermatogenesis
Source: Genes (Basel). 2020 Jan 11;11(1):84. doi: 10.3390/genes11010084 (PMC7016996; doi:10.3390/genes11010084)
Supplement: Supplementary file 1 [file genes-11-00084-s001.pdf]

Table S1. PCR-related primer list.

Gene	Primer sequence
Rybp-1stLoxP-F	CACAGAACCGGACAAGGAGT
Rybp-1stLoxP-R	TAGTGTGCAGATCATCGCATAGT
Ddx4-F	CACGTGCAGCCGTTTAAGCCGCGT
Ddx4-R	TGCCCATTCTAAACAACACCCTGAA
Ddx4-CF	CTAGGCCACAGAATTGAAAGATCT
Ddx4-CR	GTAGGTGGAAATTCTAGCATCATCC

Table S2. Real-time PCR-related primer list.

Gene name	Forward primer	Reverse primer
Ddx4	CAGAGGGTTTTCCAAGCGAGG	CCATTGCCTGAATCACTTGCT
Dazl	TCTTTGCCAGATATGGCTCAGT	CTTCTGCACATCCACGTCATTA
Rhox6	CTGGCTCAACTGCGGTACAG	ACCAATTCTGCACATCACATTCA
Mov10l1	AAACCAAAAGCTGGCCGTGA	AAATCCGACTGTCCGGCAAA
PHF7	CAGAGCCCATGCTGTAGTCA	TCTTCTCGGTTGTTGCACTG
TeX11	CAGCTGCAGTTGATCTGGATAG	GAGCAGAAGCAATACCCCAC
GAPDH	TGACATCAAGAAGGTGGTGAAGC	AAGGTGGAAGAGTGGGAGTTGCTG

Table S3. Antibody Information in this article.

Antibody	Source	Dilution	Source	Reference
Primary Antibody				
RYBP	Rabbit	1:100	Abcam	Cat.ab185971
LIN-28A	Goat	1:200	R&D systems	Cat.AF3757
γH2A.X	Mouse	1:400	Abcam	Cat.ab22551
GATA1	Rat	1:200	Santa Cruze	Cat.sc-265
SYCP1	Rabbit	1:500	Abcam	Cat.ab15090
SYCP1	Goat	1:200	Santa Cruze	Cat.sc-20837
SYCP3	Mouse	1:500	Abcam	Cat.ab97672
SYCP3	Rabbit	1:500	Abcam	Cat.ab15093
DDX4/MVH	Mouse	1:200	Abcam	Cat.ab27591
Secondary Antibody				
HRP-Goat anti Rabbit IgG		1:400	Proteintech	Cat.SA00001-2
Coralite-488-conjugated affinipure donkey anti-rabbit IgG		1:500	Proteintech	Cat.SA00013-6
Alexa-Fluor-594-conjugated donkey anti-mouse IgG		1:500	Proteintech	Cat.SA00006-7
Alexa-Fluor-488 Dnk pAb to Rat IgG		1:500	Abcam	Cat.ab150153
CY3-conjugated Affinipure Donkey Anti-Goat IgG		1:500	Proteintech	Cat.SA00009-3
Alexa-Fluor-555 Dnk pAb to Rb IgG		1:500	Abcam	Cat.ab150062
(FITC)- conjugated affinipure donkey anti-mouse IgG		1:500	Proteintech	Cat.SA000039
